# mTORC2 signalling regulates M2 macrophage differentiation in response to helminth infection and adaptive thermogenesis

**DOI:** 10.1038/ncomms14208

**Published:** 2017-01-27

**Authors:** R. W. Hallowell, S. L. Collins, J. M. Craig, Y. Zhang, M. Oh, P. B. Illei, Y. Chan-Li, C. L. Vigeland, W. Mitzner, A. L. Scott, J. D. Powell, M. R. Horton

**Affiliations:** 1 Department of Medicine, Beth Israel Deaconess Medical Center, Harvard Medical School, 330 Brooklyn Avenue, Boston, Massachusetts 02215, USA; 2 Department of Medicine, Johns Hopkins University School of Medicine, 735 North Broadway, Baltimore, Maryland 21205, USA; 3 Department of Environmental Health Sciences, Johns Hopkins Bloomberg School of Public Health, 650 North Wolfe Street, Baltimore, Maryland 21205, USA; 4 Department of Respiratory Diseases, Shanghai Pulmonary Hospital, Tongji University School of Medicine, 1239 Siping Road, Shanghai 200433, China; 5 Department of Oncology, Johns Hopkins University School of Medicine, 735 North Broadway, Baltimore, Maryland 21205, USA; 6 Department of Pathology, Johns Hopkins University School of Medicine, 735 North Broadway, Baltimore, Maryland 21205, USA; 7 W. Harry Feinstone Department of Molecular Microbiology and Immunology, Bloomberg School of Public Health, Johns Hopkins University, 650 North Wolfe Street, Baltimore, Maryland 21205, USA

## Abstract

Alternatively activated macrophages (M2) have an important function in innate immune responses to parasitic helminths, and emerging evidence also indicates these cells are regulators of systemic metabolism. Here we show a critical role for mTORC2 signalling in the generation of M2 macrophages. Abrogation of mTORC2 signalling in macrophages by selective conditional deletion of the adaptor molecule Rictor inhibits the generation of M2 macrophages while leaving the generation of classically activated macrophages (M1) intact. Selective deletion of Rictor in macrophages prevents M2 differentiation and clearance of a parasitic helminth infection in mice, and also abrogates the ability of mice to regulate brown fat and maintain core body temperature. Our findings define a role for mTORC2 in macrophages in integrating signals from the immune microenvironment to promote innate type 2 immunity, and also to integrate systemic metabolic and thermogenic responses.

## Abstract (web summary)

mTORC1 and mTORC2 are alternatively required for differentiation of T cells into Th1/Th17 or Th2 cells. Here the authors show mTORC2 signalling is also needed for IL-4-induced M2 activation with functional evidence provided by a *N. brasiliensis* infection model and cold challenge to model adaptive thermogenesis.

Macrophages can be defined by their activation state as classically activated macrophages (M1 macrophages) or alternatively activated macrophages (M2 macrophages). Using this heuristic convention, the generation of M1 macrophages is the result of stimulation with IFNγ and lipopolysaccharide (LPS)^1^ and is associated with a Th1 environment. M1 macrophages have an important function in killing intracellular pathogens^1^,^2^ and produce IL-12, IL-6, TNF and iNOS^3^. On the other hand, M2 macrophage generation results from stimulation with IL-4 (refs 4, 5) or IL-13 (refs 4, 6) and is associated with a Th2 environment^1^,^4^. M2 macrophages have been implicated in fighting parasitic infections^1^,^7^ and attenuating excessive inflammation^8^, while contributing to tissue remodelling and repair^2^,^9^. Additionally, M2 macrophages have a central role in regulating glucose tolerance and systemic metabolism^10^,^11^. Through the release of IL-10, M2 macrophages maintain insulin sensitivity in adipocytes^12^,^13^ and sustain adaptive thermogenesis by inducing thermogenic gene expression in the adipose tissue of mice exposed to cold^14^. Although we use conventional M1 and M2 definitions, we also specify the context in which the macrophages are polarized and activated, as per the latest nomenclature guidelelines^15^.

Evidence indicates that macrophages have phenotypic plasticity depending on their environment^1^,^16^ and that changes in phenotype are accompanied by alterations in metabolism^17^. mTOR (the mechanistic target of rapamycin) is a serine/threonine kinase that belongs to the phosphatidylinositol 3-kinase-related kinase protein family^18^. In yeast and mammalian cells, TOR integrates environmental cues, including growth factors and nutrient or energy availability to direct cell growth^19^,^20^. mTOR signals via two complexes, TOR complex 1 (mTORC1) and TOR complex 2 (mTORC2), and each complex has specific functions in cell signalling and metabolism^21^,^22^,^23^,^24^. Studies have shown that mTOR regulates the differentiation of T cells^25^,^26^, with mTORC1 and mTORC2 activity being required for the differentiation of T cells into Th1/Th17 and Th2 cells, respectively^25^,^26^.

A role for mTOR signalling in macrophage activation, differentiation and function seems equally likely. Evidence indicates that mTOR and its related pathways can regulate the function of dendritic cells^27^,^28^,^29^,^30^,^31^,^32^, monocytes^32^ and macrophages^33^,^34^. Although the selective deletion of mTORC1 signalling inhibits Th1 differentiation of T cells, the inhibition of mTORC1 in macrophages seems to enhance expression of M1-associated cytokines^32^,^35^. The precise role of mTORC2 signalling in macrophage differentiation and function is yet to be defined. To this end, we generated mice in which the mTORC2 adaptor protein Rictor is genetically deleted in macrophages (termed Rictor LysM mice). Our data indicate a selective role for mTORC2 signalling in the generation of M2 macrophages. Furthermore, our *in vivo* findings not only define a role for mTORC2 in integrating external cues to guide macrophage M2 differentiation and function but also substantiate the critical role of M2 macrophages in controlling thermogenesis and maintaining core body temperature.

## Results

### Generation of mTOR and Rictor null macrophages

While a number of studies have used the pharmacologic inhibitor rapamycin to examine the role of mTORC1 on M1 function^36^,^37^ (although rapamycin in high doses or for prolonged periods can also inhibit mTORC2 as well) and others have examined the consequence of hyper-active mTORC1 activity by deleting TSC1 and TSC2 on macrophage differentiation and function^34^,^38^,^39^, the role of mTORC2 in regulating macrophage activation has yet to be determined. To this end, we crossed Rictor flox mice with mice that express Cre under the control of the lysozyme (LysM-Cre) promoter. As a control, and for comparisons to delineate the unique role of mTORC2 signalling, we also crossed mTOR floxed mice with the LysM-Cre mice in order to genetically ablate both mTORC1 and mTORC2 signalling in macrophages.

**Figure 1:**
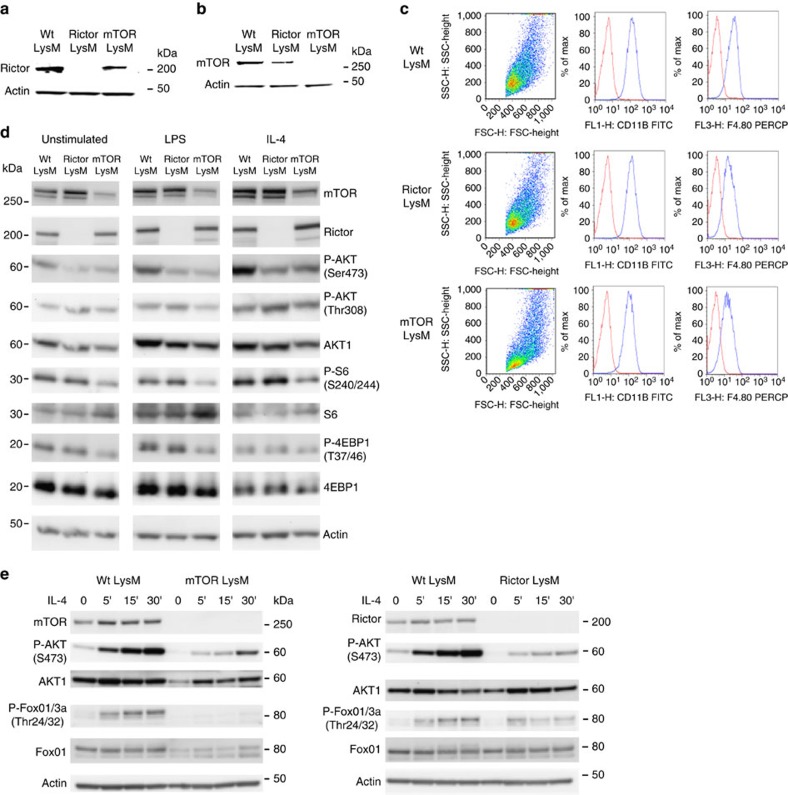
Tissue macrophage composition not affected by macrophage-specific mTOR or Rictor KO. Protein expression of Rictor (**a**) and mTOR (**b**) by BMDM. (**c**) Flow cytometry of BMDM. (**d**) Western blot of BMDM stimulated with LPS or IL-4 for 15 min. (**e**) Time course western blot of BMDM stimulated with IL-4. Experiments were performed three times with three mice per group.

First, to determine if mTOR and Rictor were efficiently genetically deleted in macrophages, bone marrow-derived macrophages (BMDM) were analysed for the synthesis of mTOR and Rictor protein using immunostained western blots. Rictor and mTOR were not detectable in lysates from the Rictor LysM and mTOR LysM macrophages, respectively (Fig. 1a,b). The selective deletion of mTOR or Rictor in macrophages did not have a substantial effect on the overall cellularity of the lungs, liver, skin or spleen (Supplementary Fig. 1a). Although the total baseline cellularity in the bone marrow of Wt LysM, mTOR LysM and Rictor LysM mice were similar, upon *in vitro* culture with MCSF1 for 7 days, there was a decrease in the yield of macrophages from the mTOR LysM bone marrow (Supplementary Fig. 1b). To test for possible cellular effects caused by lysozyme expression in other cell types^40^, we compared the numbers of alveolar macrophages, neutrophils, dendritic cells and interstitial macrophages in the lungs. There were no strain-associated differences in the total numbers or percentages of these cells (Supplementary Fig. 1c). Thus, prior to any *in vivo* activation, the tissue macrophages, dendritic cells and neutrophils were equivalent in number between the Wt LysM, mTOR LysM and Rictor LysM mice.

Next, we sought to confirm the biochemical and functional consequences of deleting Rictor and mTOR in BMDM. mTORC1 activity of LPS-stimulated cells was interrogated by examining the phosphorylation status of S6 and 4-EBP1, targets of mTORC1 (Fig. 1d). mTORC2 activity was assessed in IL-4-treated cells by measuring the phosphorylation of Akt at serine 473. As a control, we measured the phosphorylation of Akt at Threonine 308, which is not mTORC2 dependent. As predicted, the deletion of mTOR significantly reduced the phosphorylation of S6, 4EBP-1 and Akt. Alternatively, the deletion of Rictor selectively mitigated Akt phosphorylation of serine 473 while the level of phosphorylation of S6 was near that of the control (Fig. 1d). In addition, we demonstrate decreased mTORC2 activation over time in the mTOR and Rictor LysM null macrophages as measured by phosphorylation of AKT-S473 as well as the downstream target of Akt activation, P-Foxo (Fig. 1e). Thus the mTOR LysM and Rictor LysM mice harbour macrophages that are selectively defective in carrying out the biochemical functions of mTORC1/mTORC2 and mTORC2, respectively.

### mTORC2 is not required for M1 macrophage differentiation

**Figure 2:**
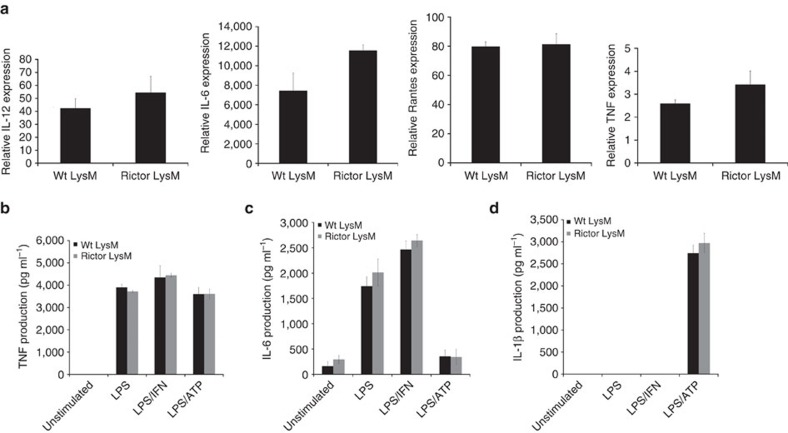
Rictor LysM macrophages have normal M1 function. (**a**) Real-time PCR analysis of M1 cytokines expression in BMDM following 24 h stimulation with LPS. (**b**) TNF production by BMDM stimulated with LPS, LPS/IFN and LPS/ATP for 24 h. (**c**) IL-6 production by BMDM stimulated with LPS, LPS/IFN and LPS/ATP for 24 h. (**d**) IL-1β production by BMDM stimulated with LPS, LPS/IFN and LPS/ATP for 24 h. Error bars represent s.d. Experiments were performed three times with four mice per group for all figures.

Pharmacologic inhibition of mTORC1 promotes the increased expression of M1 cytokine expression^36^. Because it is possible that mTORC2 was part of the mechanism that resulted in enhanced M1 activation after blocking mTORC1, we tested the role of mTORC2 activity on M1 cytokine expression. BMDM were skewed to an M1 phenotype with LPS, LPS/IFNγ or LPS/ATP and the expression of M1 markers were measured by RT-PCR or ELISA. Rictor LysM BMDM had similar expression levels of the M1-associated genes/markers IL-12α, IL-6, RANTES and TNF as that observed in Wt LysM BMDM (Fig. 2a). Rictor LysM BMDM also produced similar levels of TNF, IL-6 and IL-1β protein as measured by ELISA (Fig. 2b–d). These data suggest that mTORC2 signalling does not play a significant role in regulating M1 activation and function.

**Figure 3:**
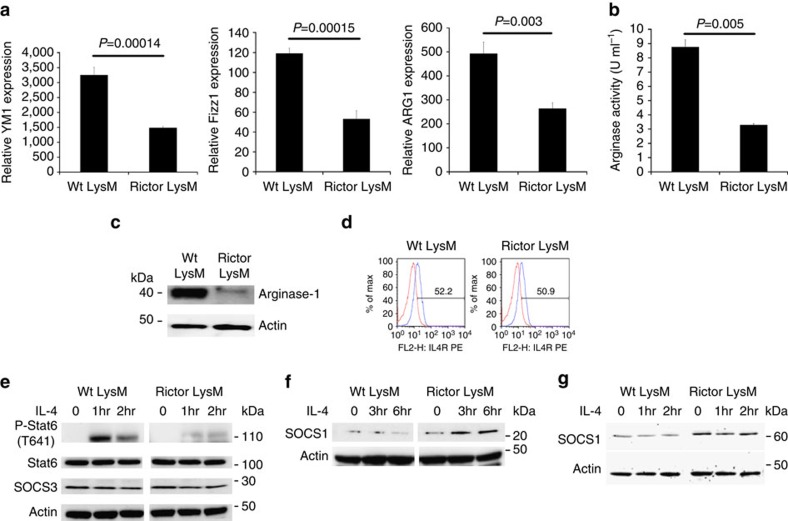
M2 skewing is defective in Rictor-deficient macrophages. (**a**) RNA expression of the M2 markers *YM1*, *Fizz1* and *Arg1* by BMDM skewed with IL-4 and restimulated with IL-4 for 24 h. (**b**) Arginase activity of BMDM skewed with IL-4 and stimulated with LPS for 24 h. (**c**) Western blot analysis of arginase-1 expression by BMDM skewed with IL-4 for 48 h. (**d**) Flow cytometry of IL-4Rα on bone marrow macrophages. (**e**) Western blot analysis of BMDM stimulated with IL-4. (**f**) Western blot analysis of SOCS1 expression by BMDM stimulated with IL-4 for 6 h. (**g**) Western blot analysis of SOCS5 expression by BMDM stimulated with IL-4 for 2 h. Error bars represent s.d. Experiments were performed three times with five mice per group. Statistical significance was determined by Student's *t*-tests performed with Bonferroni correction.

Next we wanted to determine if mTORC2 signalling played a role in the differentiation and function of M2 macrophages. To this end, BMDM were M2 polarized with IL-4 and the expression of canonical M2 markers were measured by RT-PCR. Rictor LysM BMDM had significantly lower expression of *Ym1* (Chil3), *Fizz1* (Retnla) and *Arg1* (Fig. 3a) than the M2-polarized BMDM from the control LysM mice. Consistent with these expression results, arginase enzymatic activity was significantly lower in Rictor LysM BMDM (Fig. 3b) as was the steady state levels of Arginase-1 protein (Fig. 3c). The diminished M2 activation in the Rictor LysM mice was not due to decreased expression of the IL-4 receptor, as there was no significant difference in the cell-surface expression between Wt LysM and Rictor LysM BMDM (Fig. 3d). Additionally, we observed significantly decreased levels of expression of *Arg1, Ym1* and *Mrc1* (mannose receptor 1, CD206) by mTOR LysM BMDM indicating that M2 macrophage generation occurs in an mTOR-dependent manner (Supplementary Fig. 2.)

Next we wanted to determine the mechanism by which mTORC2 regulates M2 differentiation. Previous studies have demonstrated that the deletion of Rictor in T cells inhibits Th2 differentiation in part by mitigating STAT6 phosphorylation^26^. The most studied inducer of M2 differentiation of macrophages is IL-4-induced STAT6 phosphorylation^41^. Thus, we sought to determine the effect of Rictor deletion on IL-4-mediated STAT6 phosphorylation in macrophages. BMDM were stimulated with IL-4 and the phosphorylation status of STAT6 was determined by western blotting (Fig. 3d). While IL-4 strongly induced phosphorylation of STAT6 in the control LysM macrophages, deletion of Rictor resulted in a significantly decreased level of STAT6 phosphorylation, indicating that the defect observed in the M2 differentiation of Rictor LysM macrophages was due to altered IL-4R signal transduction.

In T cells, the defect in STAT6 phosphorylation was shown to coincide with increased expression of suppressor of cytokine signalling 5 (SOCS5)^26^. Based on these data we sought to determine whether SOCS family members contributed to the observed decrease in STAT6 phosphorylation in Rictor LysM macrophages. BMDM were stimulated with IL-4 and western blot analysis was performed to estimate the levels of SOCS3 (Fig. 3e), SOCS1 (Fig. 3f) and SOCS5 (Fig. 3g). The expression of SOCS3 was unchanged and equivalent between the control LysM and Rictor LysM macrophages. However, both SOCS1 and SOCS5 had significantly increased expression in Rictor LysM macrophages, with SOCS5 being expressed regardless of stimulation with IL-4 and SOCS1 being strongly upregulated upon IL-4 stimulation. These data indicate that mTORC2 functions as an inhibitor of the synthesis of both SOCS1 and SOCS5 in macrophages, allowing for phosphorylation of STAT6, which drives M2 macrophage differentiation. Taken together these data suggest that mTORC2 plays a unique role in promoting M2, but not M1 macrophages.

### mTORC2 signalling and the response to hookworm infection

**Figure 4:**
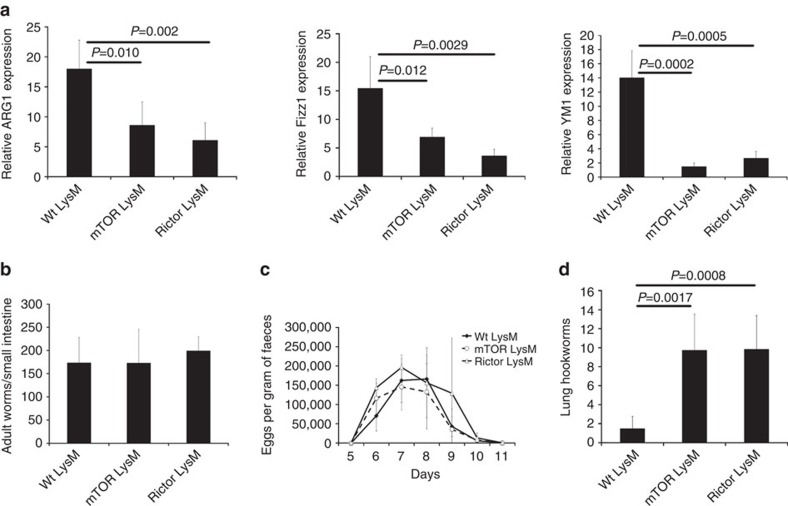
mTOR and Rictor deficiency inhibit M2 macrophage responses to hookworm infection. (**a**) Real-time PCR analysis of markers of alternative activation in lung tissue 5 days following subcutaneous injection of *Nippostrongylus brasiliensis*. (**b**) Worm burden in small intestine 5 days following subcutaneous injection of *N. brasiliensis*. (**c**) Average eggs per gram of protein in faeces following subcutaneous injection of *N. brasiliensis*. (**d**) Number of cell-free *N. brasiliensis* larvae in the lung 2 days following secondary infection. Error bars represent s.d. Experiments were performed three times with 10 mice per group. Significance determined by 1-way ANOVA followed by Tukey's test.

We wanted to determine the role of mTORC2 signalling in promoting M2-mediated immune responses *in vivo*. M2 macrophages have been shown to play a critical role in host defenses against parasitic helminths^42^,^43^ and the rodent hookworm *Nippostrongylus brasiliensis* has been extensively characterized for its ability to induce a strongly polarized Th2 immune response^44^ and robust generation of M2 macrophages in the lungs during the early, tissue migratory stage of the infection. In order to analyse the role of mTORC2 signalling in the generation of M2 macrophages following primary exposure to the parasite, control LysM, mTOR LysM and Rictor LysM mice were infected with 400 stage 3 *N. brasiliensis* larvae (L3) and 5 days later the lungs were harvested and RNA was analysed for M2-associated gene expression (Fig. 4a–c). The lungs from infected mTOR LysM and Rictor LysM mice displayed significantly reduced expression of *Arg1*, *Fizz1* and *Ym1* when compared to Wt LysM mice. Flow cytometry of whole lungs indicated similar percentages of total alveolar and interstitial macrophages after primary infection indicating that decreased levels of M2 markers were not due to decreased lung cellularity (Supplementary Fig. 3). There were no significant differences in adult worm burden in the small intestine indicating that all mice did in fact receive equivalent infections of the helminth (Fig. 4b). In addition, the magnitude and dynamics of parasite egg production was not significantly different between groups (Fig. 4c) indicating that M2 macrophages are not playing a role in blocking larval development in the lungs or in accelerating the expulsion of adult hookworms from the intestine during a primary infection. These data support an important role for TORC2 signalling in the specific generation of M2 macrophages *in vivo*.

M2 macrophages have been reported to be important for the resolution of inflammation and promoting repair after lung injury^45^.Using measures of pulmonary function, we sought to determine whether mTOR LysM and Rictor LysM mice had increased lung damage following primary infection. Mice were exposed to 400 infective stage L3 *N. brasiliensis* and 21 days later their lungs were subjected to pulmonary function testing. No significant differences were observed between Wt LysM, mTOR LysM and Rictor LysM mice when comparing diffusion capacity, lung compliance or lung resistance (Supplementary Fig. 4). These data do not support a role for M2 macrophages in the resolution of lung injury following primary hookworm infection.

In secondary hookworm infection, M2 macrophages have been shown to surround and kill hookworm larvae in the lungs, subsequently reducing the number of *N. brasiliensis* larvae that reach the gut^43^. Based on these data, we sought to determine whether Rictor LysM mice would be able to mount an effective secondary response to hookworm infection. In this model, adult worms are expelled from the intestines such that the mice are parasite-free by day 11. Wt LysM, mTOR LysM and Rictor LysM mice received a primary subcutaneous infection as outlined above, and then 4 weeks later were given a secondary intravenous infection of 1000 L3 *N. brasiliensis* larvae (to avoid larval trapping in the skin). Two days following secondary infection, the lungs were harvested, fixed and histology was performed. Lungs of Wt LysM mice contained numerous eosinophilic granulomas, with entrapped larvae and few free larvae (Fig. 4d). In contrast, the lungs from mTOR LysM and Rictor LysM mice contained free larvae in the airways with little evidence of granuloma formation (Fig. 4d). These data indicate that macrophage mTORC2 signalling is required for the effective M2 macrophage response associated with lung-based killing of hookworm larvae during secondary challenge.

### mTORC2 signalling and adaptive thermogenesis

**Figure 5:**
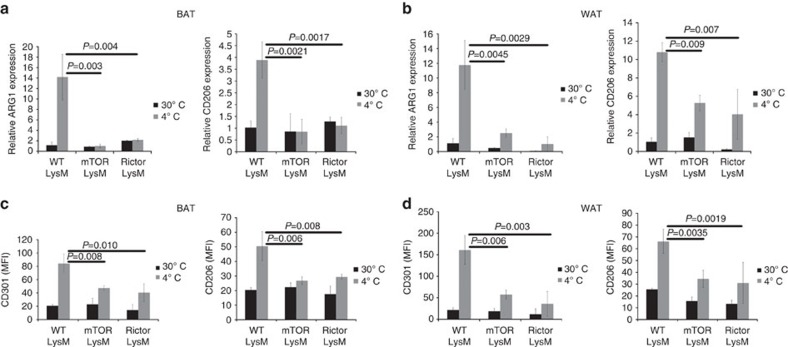
BAT and WAT macrophages have abrogated M2 function when cold challenged. Real-time PCR analysis of markers of alternative activation in BAT (**a**) and WAT (**b**) housed at 30 °C or subjected to 4 °C challenge. Expression of alternative activation markers CD301 and CD206 was monitored by flow cytometry in BAT (**c**) and WAT (**d**) macrophages housed at 30 and 4 °C. Error bars represent s.d. Experiments were performed three times with five mice per group. Statistical significance was determined by 1-way ANOVA followed by Tukey's test.

It has become increasingly clear that immune cells play an important role in regulating systemic metabolism^46^,^47^,^48^,^49^. Along these lines, it has recently been demonstrated that IL-4-induced M2 activation of macrophages is required for adaptive thermogenesis in mice^14^. Exposure to cold promotes M2 macrophage differentiation that subsequently secrete catecholamines to induce thermogenic gene expression in brown adipose tissue (BAT) and lipolysis in white adipose tissue (WAT)^14^. In light of our data demonstrating defective M2 macrophage differentiation *in vitro* and *in vivo* in the absence of Rictor, we wanted to determine if mTORC2 signalling in macrophages was required to regulate thermogenesis *in vivo*. To accomplish this, we analysed the expression of M2 markers on BAT- and WAT-derived macrophages in mice housed at 30 °C (thermoneutrality) or after an acute challenge at 4 °C. Consistent with previous findings, there was an increase in the expression of *Arg1* and *Mrc1* in both BAT and WAT of wild type mice exposed to 4 °C (Fig. 5a,b). In contrast, mTOR LysM and Rictor LysM mice failed to demonstrate an increase in M2 activation of BAT and WAT macrophages when exposed to the cold environment, as demonstrated by the lack of expression of *Arg1* and *Mrc1*. Brown fat macrophages also failed to express the M1-associated genes CD274, NOS2 and TNF, indicating that brown fat macrophages from mTOR LysM and Rictor LysM are not preferentially becoming M1 in the absence of M2 gene expression (Supplementary Fig. 5). Regardless of genotype, all mice demonstrated an increase in the percentage of macrophages in the brown fat following 6 h at 4 °C (Supplementary Fig. 6). This increase appeared to be due to increased influx from the periphery since the expression of the proliferation marker Ki67 was unchanged (Supplementary Fig. 7). When we analysed the surface expression of the M2 surface markers CD301 (the C-type lection family member CLEC10A) and CD206 (mannose receptor) by flow cytometry, we observed a significant reduction in their expression on BAT- and WAT-derived macrophages from mTOR LysM and Rictor LysM mice exposed to 4 °C (Fig. 5c,d). Overall, these data support a role for TORC2 signalling in the generation of M2 macrophages *in vivo* in response to systemic thermoregulatory mechanisms.

**Figure 6:**
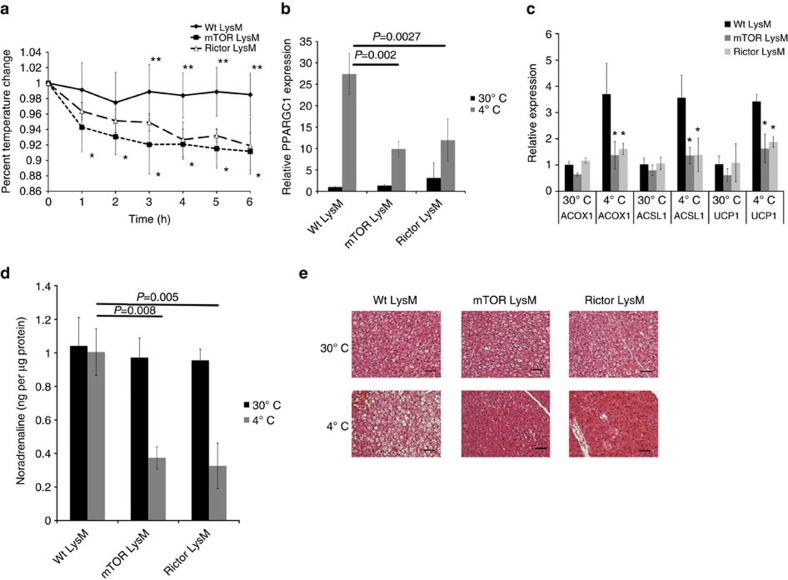
mTOR LysM and Rictor LysM mice have a defect in adaptive thermogenesis. (**a**) Core body temperature of mice during cold challenge at 4 °C over 6 h. *indicates statistical significance between Wt LysM and Rictor LysM. **indicates statistical significance between Wt LysM and mTOR LysM. (**b**,**c**). Real-time PCR analysis of thermogenic genes in BAT from mice housed at 30 °C or subjected to 4 °C challenge for 6 h. (**d**) Noradrenaline content of BAT at 30 °C or subjected to 4 °C challenge for 6 h. (**e**) Representative histology (haematoxylin and eosin staining) of BAT at 30 °C or subjected to 4 °C challenge for 6 h, scale bars represent 50 μm. Error bars represent s.d. Experiments were performed three times with eight mice per group. Statistical significance was determined by either Student's *t*-test or 1-way ANOVA followed by Tukey's test. *=statistical significance where *P*<0.05.

To investigate the effect of defective M2 macrophage generation on cold-induced thermogenesis in intact animals, we challenged Wt LysM, mTOR LysM and Rictor LysM mice to 4 °C. Unlike the control LysM mice, mTOR LysM and Rictor LysM mice exhibited a drop in core body temperature when exposed to 4 °C (Fig. 6a). In addition, the mTOR LysM and Rictor LysM mice lost significantly less weight over the 6-h cold challenge, indicating a defective thermogenic response (Supplementary Fig. 8). Wt LysM mice responded to the decrease in temperature by expressing the thermogenic genes *Ppargc1a* and *Ucp1* and the β-oxidation genes *Acox1* and *Acsl1* in BAT (Fig. 6b,c)^50^,^51^. The induction of thermogenic genes by BAT was significantly abrogated in mTOR LysM and Rictor LysM mice. We also observed abrogated expression of thermogenic genes by WAT from mTOR LysM and Rictor LysM animals (Supplementary Fig. 9). These data indicate that differentiation of BAT- and WAT-resident macrophages to M2 is required for the thermogenic response to cold temperatures.

It has recently been demonstrated that thermogenic gene induction is dependent upon the production of catecholamines by BAT-resident M2 macrophages^14^. Therefore, we sought to determine if the observed defect in adaptive thermogenesis by mTOR LysM and Rictor LysM mice was due to a lack of catecholamine production by BAT-resident macrophages. After mTOR LysM, Rictor LysM and control mice were cold challenged at 4 °C for 6 h, brown fat was isolated and the concentration of noradrenaline was measured. In contrast to the control LysM mice that showed no significant change in noradrenaline whereas the mTOR LysM and Rictor LysM mice had a significant reduction in noradrenaline content when cold challenged indicating that the defective thermogenic response in these mice may be due to insufficient catecholamine production by BAT-resident macrophages (Fig. 6d).

Catecholamines released by M2 macrophages in the WAT bind the β-adrenergic receptor on adipocytes and induce the expression of tyrosine hydroxylase. This induces lipolysis of WAT and results in the release of free fatty acids into the blood stream^14^. Since we observed decreased catecholamine production in both mTOR LysM and Rictor LysM mice we sought to determine if this resulted in defective lipolysis. In order to confirm equivalent expression of the β-adrenergic receptor on adipocytes, WAT was isolated and western blot was performed for the β-adrenergic receptor (Supplementary Fig. 10). β-adrenergic receptor expression was found to be present and not significantly different between Wt LysM, mTOR LysM and Rictor LysM mice. In order to examine lipolysis we exposed Wt LysM, mTOR LysM and Rictor LysM mice to 6-h 4 °C challenge, isolated white fat and performed flow cytometry for tyrosine hydroxylase (Supplementary Fig. 11a). Wt LysM mice had a significant increase in the MFI of tyrosine hydroxylase when challenged at 4 °C for 6 h; however both mTOR LysM and Rictor LysM mice demonstrated no increase in the MFI of tyrosine hydroxylase when cold challenged. In addition, when we measured serum-free fatty acids after 6-h cold challenge mTOR LysM and Rictor LysM had significantly less serum FFAs when compared to Wt LysM following cold challenge (Supplementary Fig. 11b). Consistent with this finding, histology revealed that mTOR LysM and Rictor LysM mice had exhausted their lipid stores in their BAT (Fig. 6e and Supplementary Fig. 12) indicating that the observed defective adaptive thermogenesis was a direct consequence of insufficient free fatty acid mobilization due to reduced catecholamine production by WAT-resident M2 macrophages.

**Figure 7:**
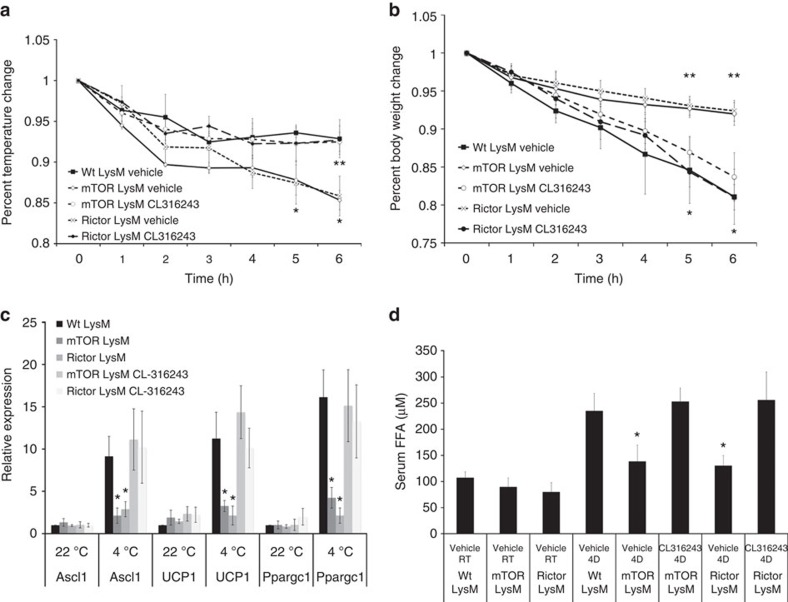
β-adrenergic receptor agonist rescues mTOR LysM and Rictor LysM mice. (**a**) Body temperature change during 6-h cold challenge. (**b**) Body weight change during 6-h cold challenge. *indicates statistical significance between Wt LysM and Rictor LysM. **indicates statistical significance between Wt LysM and mTOR LysM. (**c**) RNA expression of thermogenic genes Ascl1, UCP1 and Ppargc1 by WAT following 6-h cold challenge. (**d**) Serum-free fatty acids following 6-h cold challenge. Error bars represent s.d. All experiments were performed twice with 10 mice per group. Statistical significance was determined by 1-way ANOVA followed by Tukey's test. *= statistical significance (*P*<0.05).

These data led us to investigate whether the β-adrenergic receptor agonist CL-316243 could rescue the observed defect in thermogenesis in mTOR LysM and Rictor LysM mice. Mice were given a single injection of CL-316243 and 30 min later challenged at 4 °C for 6 h. mTOR LysM and Rictor LysM mice that received CL-316243 maintained body temperature similar to Wt LysM whereas mice that received vehicle lost significantly more body temperature (Fig. 7a). In addition, mice that received CL-316243 lost more body weight than mice that received vehicle, indicating that the thermogenic response was functional (Fig. 7b). mTOR LysM and Rictor LysM mice receiving vehicle had significantly abrogated expression of thermogenic genes in WAT; however, mice provided with CL-316243 expressed thermogenic genes to similar levels as Wt LysM mice (Fig. 7c). Treatment with CL-316243 also returned serum FFA levels to those seen in Wt LysM mice (Fig. 7d). These data indicate that the defect in thermogenesis observed in mTOR LysM and Rictor LysM mice is due to defective M2 macrophage generation that in turn abrogates the β-adrenergic response. Overall, our data support an essential role for mTORC2 signalling in the *in vivo* differentiation of M2 macrophages and indicate that mTOR signalling is necessary for proper adaptive thermogenesis.

## Discussion

Macrophages have traditionally been classified as either classically activated (M1) or alternatively activated (M2) depending upon their stimulatory environment^5^,^52^,^53^. However, the precise mechanism by which macrophages differentiate into the M1 and M2 phenotypes remains incompletely understood. We have used a novel Rictor LysM mouse model to demonstrate for the first time that mTORC2 regulates the differentiation and key functional abilities of M2 macrophages. Further, we provide evidence that mTORC2 has no demonstrable effect on the course of M1 differentiation. Our data clearly demonstrate a critical role for mTORC2 in regulating M2 macrophage differentiation *in vitro* and *in vivo* in response to both infection and systemic thermogenic signals. We propose that mTORC2 integrates multiple signals from the immune microenvironment to guide this differentiation. Along these lines we demonstrate that the decrease in IL-4 mediated signalling in Rictor LysM null macrophages is associated with an increase in the inhibitors SOCS1 and SOCS5. While this represents one mechanism by which mTORC2 specifically regulates macrophage differentiation, given the multitude of signalling pathways controlled by mTOR we suspect that other mechanisms are likely involved as well. While the LysM-Cre model has been extensively employed to examine macrophage function *in vivo*^14^,^34^,^38^, it is important to note that LysM is also upregulated in neutrophils and dendritic cells during inflammation and we cannot exclude the possibility that deletion of mTORC2 in these cells could have had some effect on the *in vivo* phenotypes presented here.

Previous studies have examined the role of TSC1 in regulating macrophage differentiation and function. Deleting TSC1 in macrophages using LysM-Cre leads to hyperactive mTORC1 activity and cells that increase M1-associated cytokine production and are refractory to IL-4-induced M2 macrophage differentiation^34^. Subsequently it was demonstrated that TSC1 inhibits M1 macrophage polarization in an mTORC1-independent fashion while promoting M2 properties in an mTOR-dependent CCAAT/enhancer-binding protein-β pathway^38^. In addition, mTORC1 signalling has been shown to influence Ac-CoA synthesis and histone acetylation of several M2 genes^54^. These reports suggest that, while mTORC2 regulates M2 macrophage differentiation *in vitro* and *in vivo*, mTORC1 signalling is not completely dispensable for expression of a number of genes critical for M2 function.

There is evidence that mTOR and its related pathways regulate the production of cytokines such as IL-12 and IL-10 in dendritic cells^27^,^28^,^29^,^30^,^32^, cytokine production of macrophages challenged by *Mycobacterium tuberculosis*^33^, and differentiation of T cells^26^,^55^. T cells lacking both mTORC1 and MTORC2 activity are unable to develop into Th1, Th2 or Th17 cells; rather, they become CD4^+^ T regulatory cells^25^. CD4^+^T cells lacking only mTORC1 activity are unable to become Th1 or Th17 cells; however, they are capable of becoming Th2 cells^25^. T cells lacking mTORC2 activity are unable to become Th2 cells, while maintaining their ability to develop into Th1 or Th17 cells^26^. We have demonstrated that mTORC2 regulates M2 differentiation and function in a way that is analogous to its role in Th2 differentiation. By providing a gate for both Th2 (ref. 26) and M2 differentiation, mTORC2 is strategically positioned to be the primary regulator of macrophage polarization in settings where Th2-mediated inflammation is dominant such as parasitic helminth infections, allergic responses, and tissue repair and wound healing^56^,^57^.

In addition to their role as primary effectors of the innate immune response, macrophages, and in particular, M2 macrophages, have been shown to play an integral role in adaptive thermogenesis^14^. Nguyen *et al*. have demonstrated that exposure to cold results in an upregulation of BAT M2 macrophages, and that these macrophages induce the expression of thermogenic genes within brown fat^14^. In their model, mice deficient in IL-4/IL-13 signalling were unable to upregulate adipose tissue M2 macrophages and demonstrated impaired thermoregulation during cold exposure^14^. Similarly, we have found that exposure to cold induces the expression of BAT M2 macrophages, and that this expression is severely impaired in the Rictor LysM and mTOR LysM mice and is associated with impaired thermoregulation. The mice used in our study had no defect in IL-4/IL-13 signalling, thus supporting the idea that adaptive thermogenesis is specifically dependent upon fully functional M2 macrophages. The observation that selectively deleting mTORC2 activity in macrophages results in such profound defects in the ability of BAT and WAT to defend their core body temperature serves to underscore the critical role of M2 macrophages and mTORC2 signalling in regulating systemic thermogenesis and as a critical component of an evolutionarily conserved environmental sensing system.

Overall our data demonstrate that mTORC2 regulates M2 macrophage differentiation and function, while being dispensable for M1 differentiation. Our data identify mTORC2 as a critical sensor that enables M2 macrophages to respond to helminth infection as well as regulated systemic metabolism. M2 macrophages play a key role in the elimination of various infections, and they have been implicated in diseases involving the lungs^1^,^7^,^58^,^59^, heart^1^,^60^,^61^, central nervous system^62^,^63^, and in the formation of atherosclerotic plaques^64^,^65^. As such our findings have broad implications for both infectious and non-infectious disease. Indeed, by integrating environmental cues, mTORC2 signalling connects the diverse role of M2 macrophages in regulating infection and thermogenesis. As a result, various steps in the mTORC2 signalling pathway may ultimately prove to be novel therapeutic targets for altering the course of systemic disease.

## Methods

### Mice

All experimental procedures were approved by the Animal Care and Use Committee of Johns Hopkins University and mice were maintained in accordance with their guidelines. C57BL/6 Wt LysM mice, Rictor flox mice and mTOR flox mice were obtained from Jackson Laboratory (Bar Harbor, ME). All experiments were conducted using either 8-week-old female or male mice for a given experiment.

### Generation of BMDMs

Macrophage precursors were obtained from the bone marrow of each individual mouse line and differentiated on untreated polystyrene Petri dishes (Fisher Scientific, USA) in 10 ml of Roswell Park Memorial Institute media (RPMI) supplemented with 10% FBS, 1% penicillin/streptomycin and 2 mM L-glutamine (cell media) plus 20% L929-conditioned media containing M-CSF^66^. The cells were incubated at 37 °C with 5% CO_2_. On day 4, the cell media and any non-adherent cells were removed; adherent cells were washed with PBS before the addition of 10 ml of fresh cell media plus 20% L929 media. On day 7, macrophages were washed with PBS and then incubated with 10 ml of Cellstripper (Mediatech, Manassas, VA) for 45 min prior to gentle pipette agitation. Cell solutions were spun down and suspended in RPMI+10% FBS, 1% penicillin/streptomycin and 2 mM L-glutamine. For skewing, bone marrow macrophages were skewed to M1 with IFNγ (500 U ml^−1^) or to M2 with IL-4 (20 ng ml^−1^) for 48 h.

### Cell stimulation

BMDMs were plated in 24-well plates at a concentration of 1 million cells per well or in 96-well plates at a concentration of 250 K cells/well and allowed to adhere overnight in cell media. The following day, the cell media was removed and adherent cells were washed with PBS before the addition of cell media lacking FBS. Cells were then stimulated with IL-4 (20 ng ml^−1^) (Peprotech), LPS (50 ng ml^−1^) (Sigma) or IFNγ (500 U ml^−1^) (Sigma) for 24 h. Supernatant was collected and stored at −80 degrees for future ELISA, arginase assay and nitrite assay experiments. Cells were processed for RNA extraction as detailed below.

### RNA extraction and quantitative real-time RT-PCR

Total cell RNA was extracted using TRIzol reagent (Invitrogen, Carlsbad, CA) and reversed transcribed by Superscript as per the manufacturer's protocol (Invitrogen). Real-time PCR was performed on an Applied Biosystems 7300 PCR machine using Applied Biosystems reagents (Carlsbad, CA) and normalized to 18s rRNA. Values were calculated using the delta Ct method in reference to unstimulated WT LysM samples for each primer. All primers and probe sets used were purchased from Applied Biosystems (Carlsbad, CA). IL12a (Mm00434165), IL6 (Mm00446190), Rantes (Mm01302427), TNF (Mm00443258), YM1 (Mm00657889), Fizz1 (Mm00445109), Arg1 (Mm00475998), CD206 (Mm00485148), Ppargc1 (Mm00504720), Acox1 (Mm01246831), Ascl1 (Mm00484217), UCP1 (Mm01244861), Cd274 (Mm00452054), NOS2 (Mm01309897), Ki67 (Mm01278617).

### ELISA and arginase assays

Cytokine expression was measured via enzyme-linked immunosorbent assay (ELISA) on a Bio-Rad (Hercules, CA) plate reader and analysed with Microplate Manager III (Bio-Rad) software. ELISA kits for TNF were from eBioscience (San Diego, CA). Arginase activity in culture supernatants was measured using a QuantiChrom arginase assay kit according to the manufacturer's specifications (DARG-200, BioAssay Systems, Hayward, CA). Serum FFA ELISA kit was purchased from Abcam (Cambridge, UK).

### Thermogenesis experiments

For cold challenge experiments mice were individually housed in cages that had been pre-chilled at 4 °C. Core body temperature was monitored hourly by rectal temperature probe. For thermoneutrality experiments, mice were adapted to 30 °C for 3 weeks before experimentation. Tissues were collected at the end of a 6-h cold challenge, and processed for RNA and norepinephrine analysis.

### FACS analysis

Adipose tissues were minced and digested with collagenase I (3 mg ml^−1^, Gibco) for 45 min at 37 °C in a shaker. The digested cell suspension was centrifuged at 1,600 r.p.m. for 5 min to separate stromal-vascular fraction from adipocytes. Pelleted cells were re-suspended in FACS buffer (PBS containing 5% FBS and 1% gluatamine) and passed through a 40 μm strainer to remove large cellular debris. FACS analysis was performed on a BD FACS Caliber or FACS Celesta. Antibodies used are listed in Supplementary Fig. 13. Lung FACS gating strategy is displayed in Supplementary Fig. 14.

### Western blotting

Cells were plated at a concentration of 3 million cells per 6-well plate and incubated overnight in cell media without FBS. The following day, cells were stimulated with LPS or IL-4 as outlined above. Cell pellets were lysed and 20 μg of lysate was fractionated by SDS-PAGE and transferred to nitrocellulose. Membranes were then blocked with 5% bovine serum albumin, washed, and incubated with primary antibodies (1:2,000) (Cell Signaling Technology, Danvers, MA) overnight at 4 °C. Membranes were then washed and incubated for 1 h with anti-rabbit IgG horseradish peroxidase linked secondary antibody (1:5,000) (ECL, Buckinhamshire, UK) prior to developing with a chemiluminescent system (Amersham, Buckinhamshire, UK). Original immunoblots are shown in Supplementary Fig. 15.

### Catecholamine measurement

Catecholamines (LDN Labor Diagnostika Nord GmbH & Co) were quantified in duplicate as per the manufacturer's protocols. Tissues were homogenized in homogenization buffer (0.01 N HCL, 0.1 mm EDTA, 4 mm NA_2_S_2_O_5_) and cellular debris was pelleted by centrifugation. The cleared homogenates were collected and stored at −80 °C before quantification. All samples were normalized to total tissue protein concentration.

### *N. brasiliensis* infection

Maintenance and enumeration of *N. brasiliensis* was performed as previously described^67^. Briefly, propagation of *N. brasiliensis* was performed by subcutaneous injection of mice with L3 larvae and isolation of faeces on days 7–10. Eggs of *N. brasiliensis* were cultured on granulated charcoal and sphagnum moss in order to isolate larvae. To initiate infection, mice were injected subcutaneously at the nape of the neck with 400 infective L3 stage larvae suspended in 200 μl of PBS. Faeces of infected mice were isolated on days 5–11, softened in water and counted.

### Diffusion factor for carbon monoxide measurement

To assess overall functional changes in the lungs following helminth-induced injury, measurement of the diffusion factor for carbon monoxide (DFCO) was performed as previously described^68^. Briefly, mice were anesthetized with a mixture of ketamine (100 mg kg^−1^)/xylazine (15 mg kg^−1^) via intraperitoneal injection. Once sedated, a tracheostomy was performed, and an 18-gauge cannula was inserted. Lungs were quickly inflated with a 0.8 ml gas mixture (0.5% neon, 1% CO and balance air), and after a 9-s breath hold, the gas was quickly withdrawn from the lungs and diluted to 2 ml with room air. The neon and CO concentrations in the diluted air were measured by gas chromatography (INFICON, Model 3000A; Inficon Inc., East Syracuse, NY) to calculate the DFCO.

### Pulmonary mechanics measurements

After DFCO assessment, mice were connected to a flexi-VentTM ventilator (Scireq, Montreal, QC, Canada) and ventilated with a tidal volume of 0.2 ml of 100% oxygen at a rate of 150 Hz with a positive end-expiratory pressure of 3 cm H_2_O. Mice were paralyzed with an i.p. injection of succinylcholine (75 mg kg^-1^), subjected to deep inspiration at 30 cm H_2_O for 5 s and returned to normal ventilation for 1 min. Baseline measurements of respiratory system resistance (Rrs), compliance (Crs) and elastance (Ers) were measured during a 2-s breath hold with a 2.5-Hz sinusoidal oscillation using the single compartment^69^. The impedance of the respiratory system was also obtained using a constant phase model to provide measurements of airway resistance (Raw), tissue damping (G) and tissue elastance (H)^70^.

### Statistical analysis

All experiments were performed in biological triplicate and results represent the mean ± s.d. All experiments were replicated at least three times. Statistical analysis was performed using either 1-way ANOVA followed by Tukey's test or a paired Student's *t*-test; *P*<0.05 was considered statistically significant.

### Data availability

The data that support the findings of this study are available from the corresponding author upon request.

## Additional information

**How to cite this article:** Hallowell, R. W. *et al*. mTORC2 signalling regulates M2 macrophage differentiation in response to helminth infection and adaptive thermogenesis. *Nat. Commun.* **8,** 14208 doi: 10.1038/ncomms14208 (2017).

**Publisher's note:** Springer Nature remains neutral with regard to jurisdictional claims in published maps and institutional affiliations.

## Supplementary Material

Supplementary Information Supplementary Figures
