# Supplementary material for: mTORC2 signalling regulates M2 macrophage differentiation in response to helminth infection and adaptive thermogenesis
Source: Nat Commun. 2017 Jan 27;8:14208. doi: 10.1038/ncomms14208 (PMC5290163; doi:10.1038/ncomms14208)
Supplement: Supplementary Information — Supplementary Figures [file ncomms14208-s1.pdf]

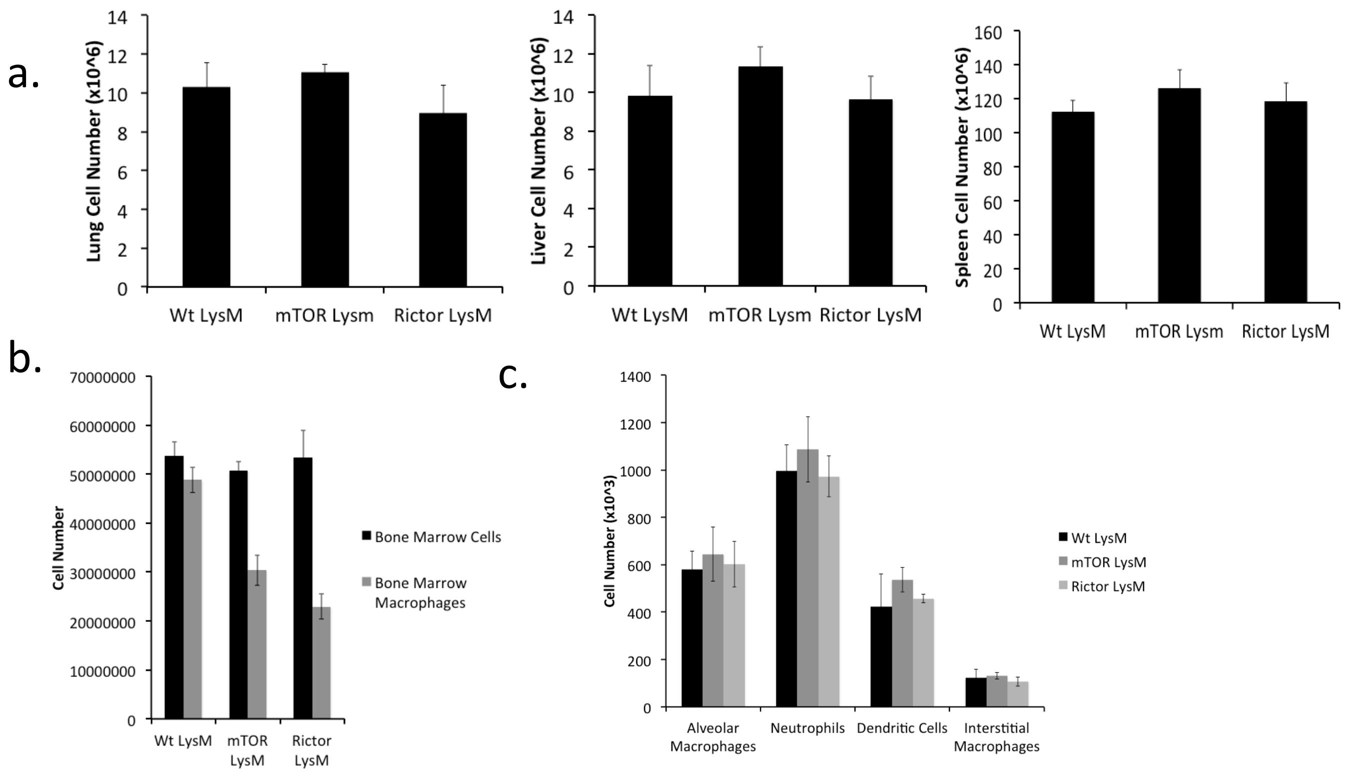

Supplementary Figure 1. mTOR LysM and Rictor LysM mice have normal cellularity and percentages of hematopoetic cells. a. Cell numbers of lung, liver, and spleen. **b.** Cell numbers of bone marrow and bone marrow derived macrophages. **c.** Cell numbers of lung alveolar macrophages, neutrophils, dendrici cells, and interstitial macrophages. Error bars represent standard deviation. All experiments were performed three times with three mice per group.

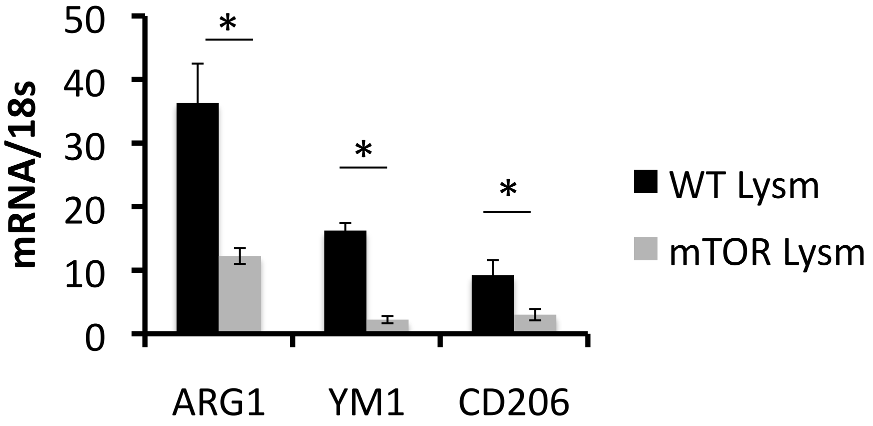

Supplementary Figure 2. mTOR LysM macrophages fail to generate M2 macrophages. RNA expression of the M2 markers *YM1*, *Fizz1*, and *Arg1* by bone marrow derived macrophages skewed with IL4 and restimulated with IL4 for 24hrs. Error bars represent standard deviation. Experiments were performed three times with five mice per group. Statistical significance was determined by Students-t tests performed with Bonferroni correction. *= statistical significance where $p<.05$.

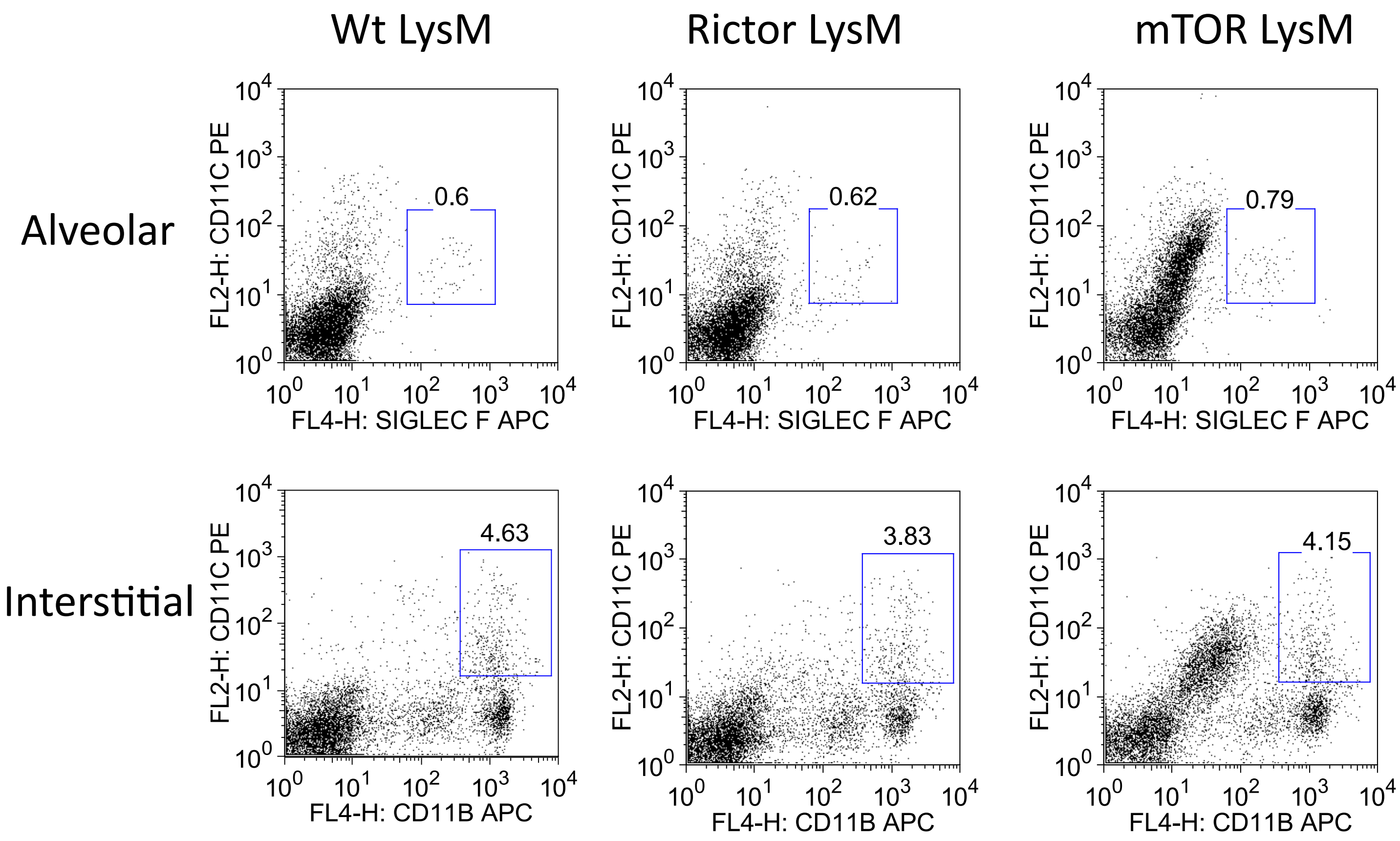

Supplementary Figure 3. mTOR LysM and Rictor LysM mice have similar numbers of lung macrophages following hookworm infection. Flow cytometry of lungs 5 days following subcutaneous injection of *Nippostrongylus brasiliensis*. Experiments were performed three times with ten mice per group.

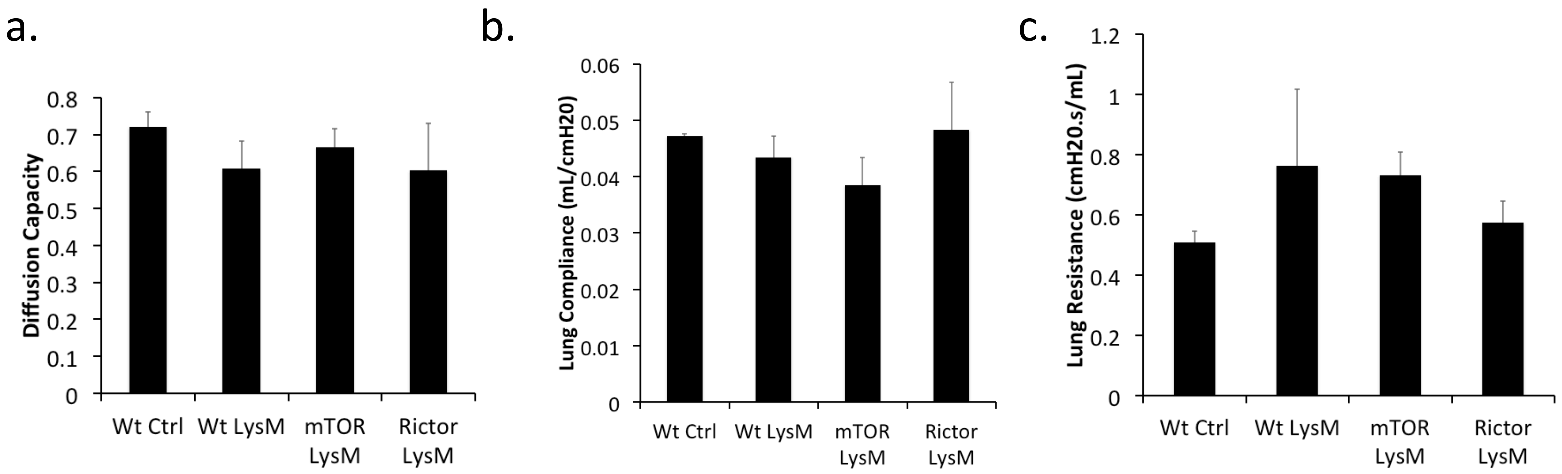

Supplementary Figure 4. mTOR LysM and Rictor LysM mice do not have increased lung damage following hookworm infection. a. Diffusion capacity of mice 21 days following hookworm infection. **b.** Lung compliance of mice 21 days following hookworm infection. **c.** Lung resistance of mice 21 days following hookworm infection. Error bars represent standard deviation. Experiments were performed three times with eight mice per group.

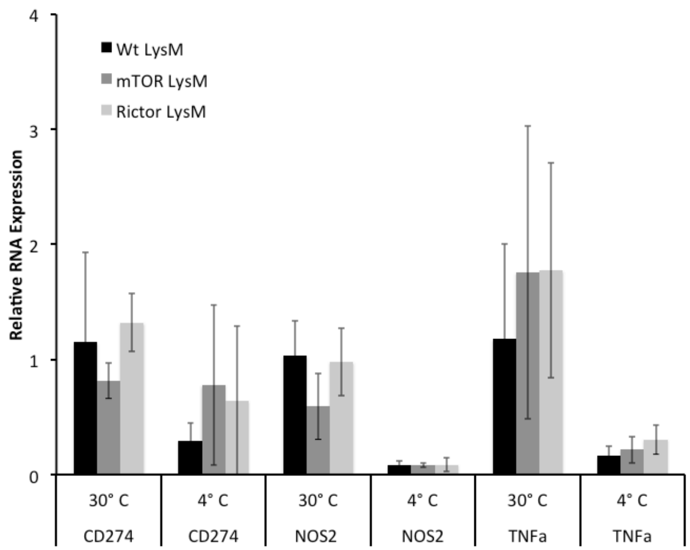

Supplementary Figure 5. mTOR LysM and Rictor LysM brown fat macrophages do not express M1 genes. RNA analysis of M1 genes in BAT following six-hour cold challenge. Error bars represent standard deviation. Experiments were performed three times with four mice per group.

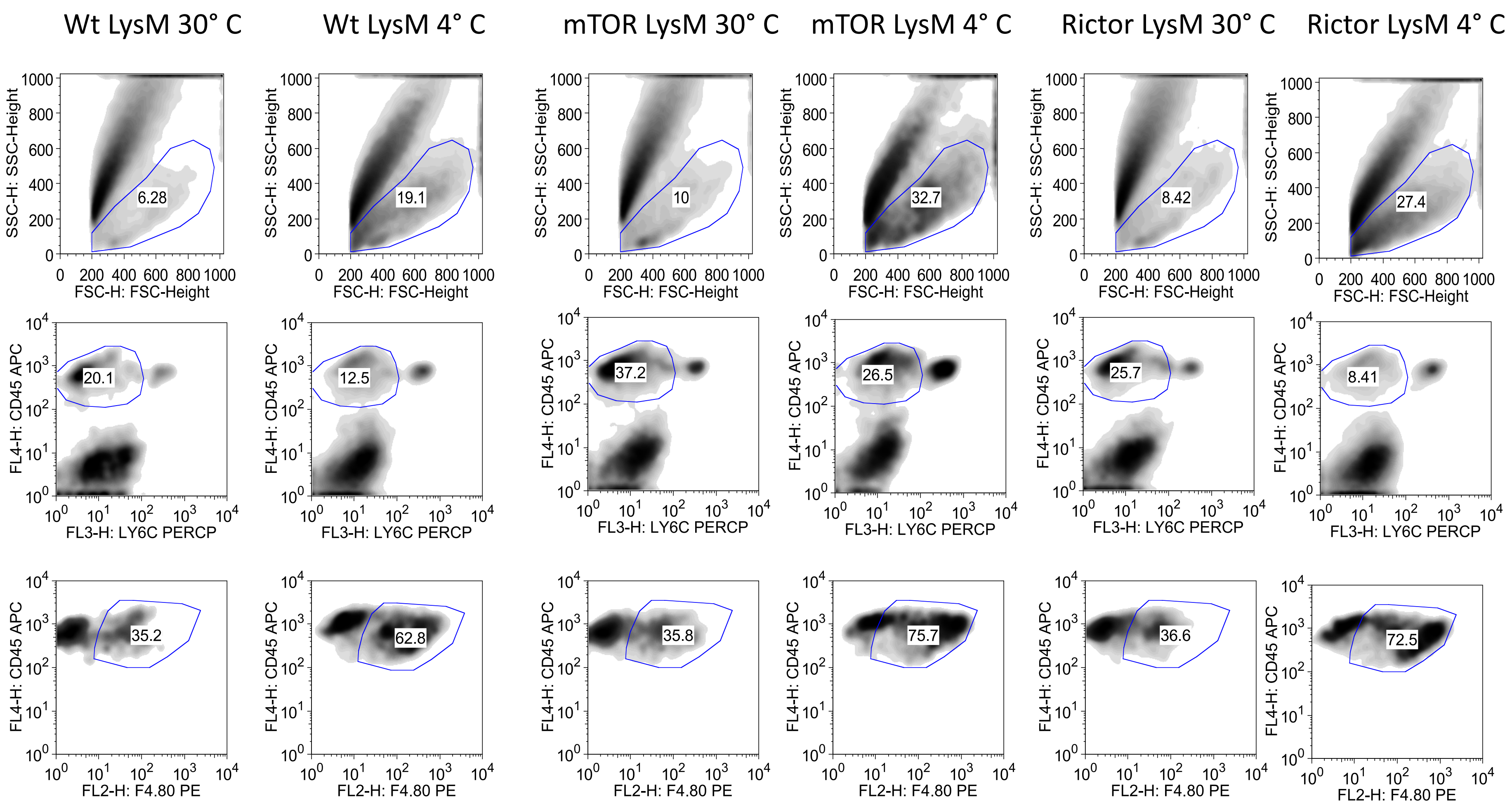

Supplementary Figure 6. mTOR LysM and Rictor LysM mice have normal percentages of brown fat macrophages. Representative flow cytometry of Wt LysM, mTOR LysM, and Rictor LysM Brown fat Macrophages. Experiments were performed three time with five mice per group.

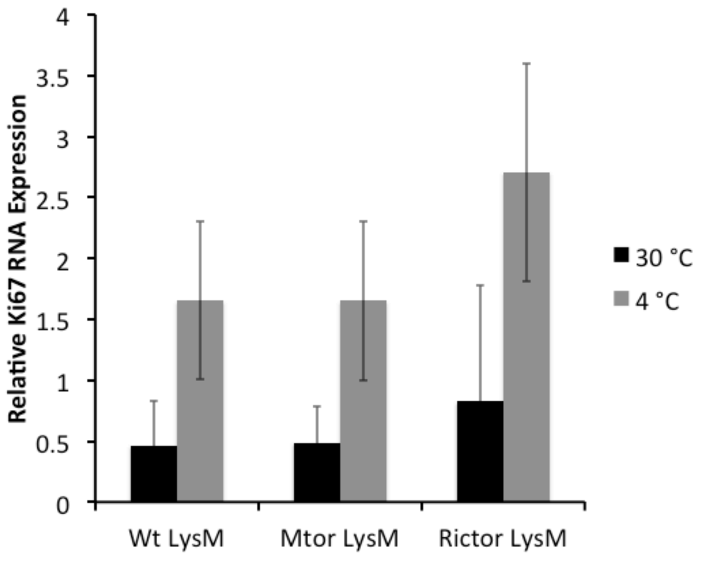

Supplementary Figure 7. Brown fat macrophages do not proliferate during six-hour cold challenge. RNA Expression of Ki67 by Brown Fat following six-hour cold challenge. Error bars represent standard deviation. Experiments were performed three times with four mice per group.

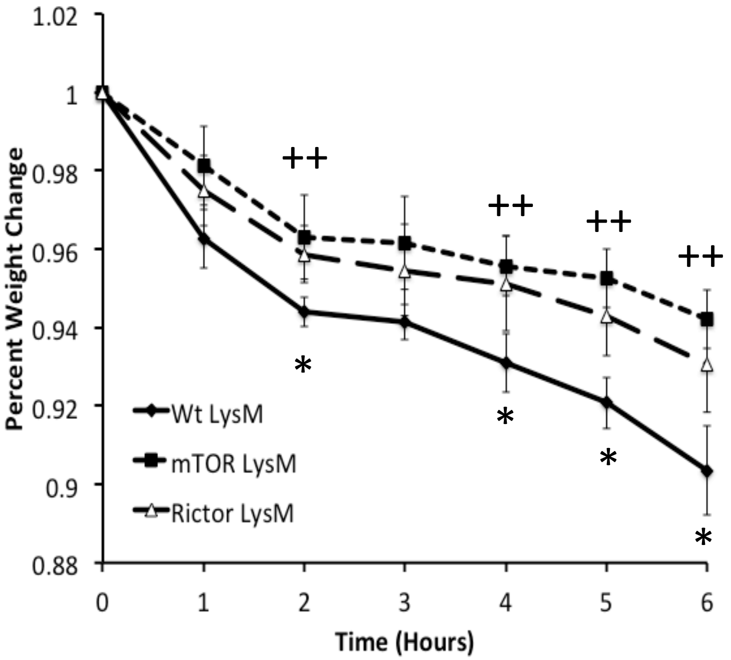

Supplementary Figure 8. mTOR LysM and Rictor LysM mice lose less weight during cold challenge. Weight change during six-hour cold challenge of Wt LysM, mTOR LysM, and Rictor LysM. Error bars represent standard deviation. Experiments were performed three times with eight mice per group. Statistical significance was determined by 1-way ANOVA followed by Tukeys test. * and ++ = statistical significance ($p<.05$)

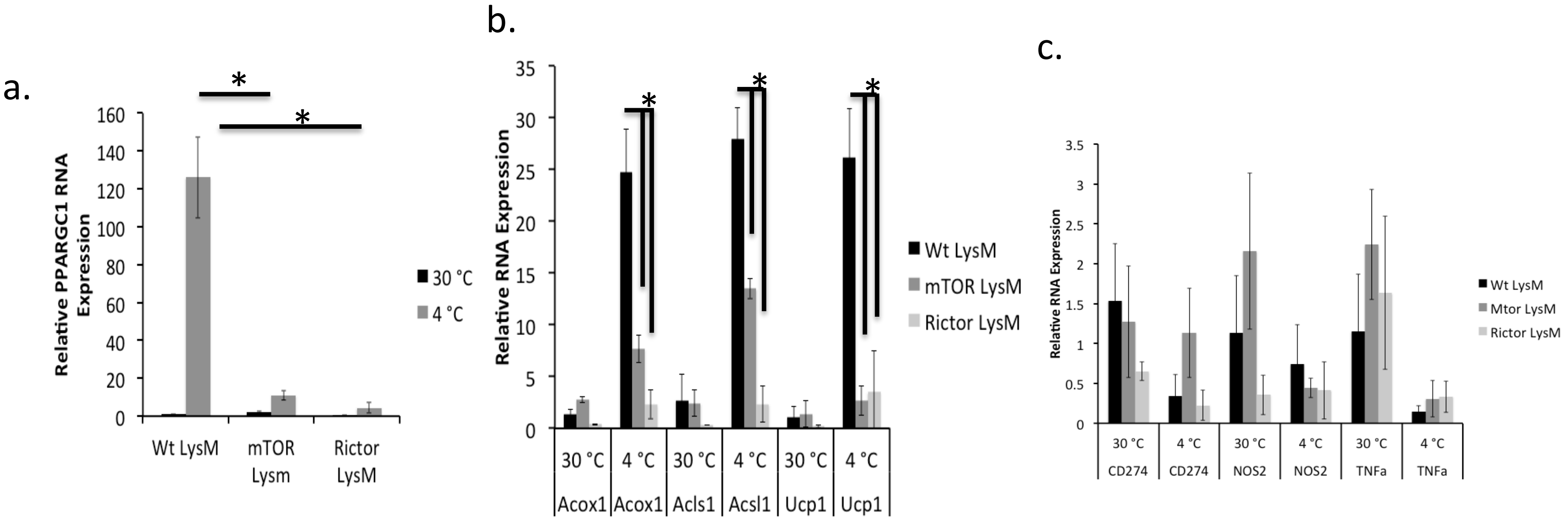

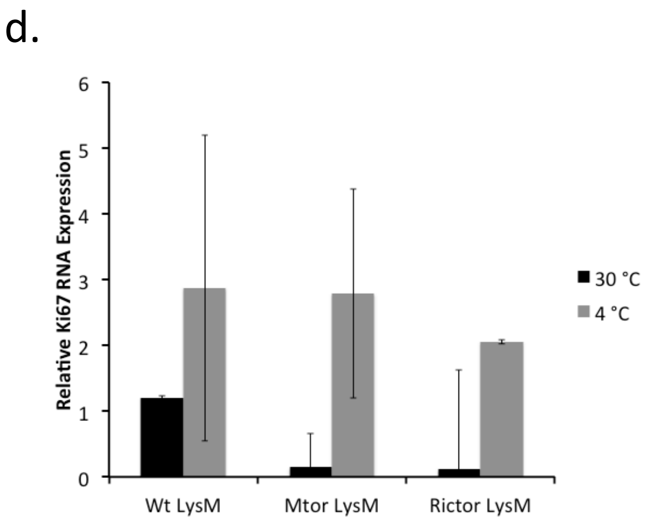

Supplementary Figure 9. mTOR LysM and Rictor LysM mice fail to upregulate thermogenic genes in WAT. a. RNA expression of Ppargc1 by WAT. **b.** RNA expression of Acox1, Acsl1, and UCP1 by WAT. **c.** M1 gene expression by WAT macrophages. **d.** Ki67 RNA Expression of WAT macrophages. Error bars represent standard deviation. Experiments were performed three times with five mice per group. Statistical significance was determined by 1-way ANOVA followed by Tukeys test. *= statistical significance (p<.05)

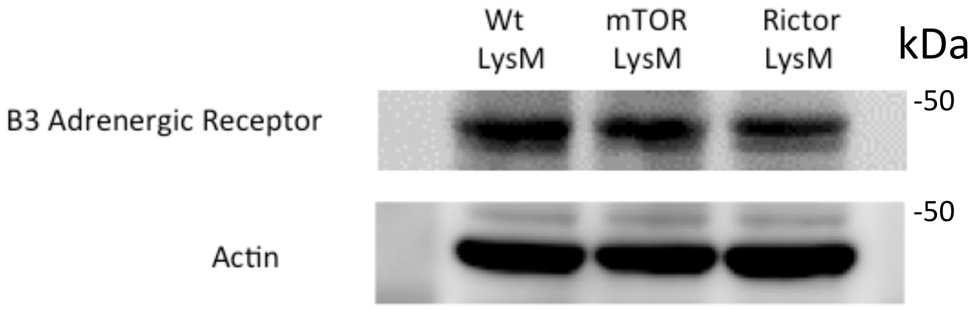

Supplementary Figure 10. mTOR LysM and Rictor LysM adipocytes express the β-adrenergic receptor. Western blot analysis of the β-adrenergic receptor from white fat adipocytes. Experiments were performed two times with three mice per group.

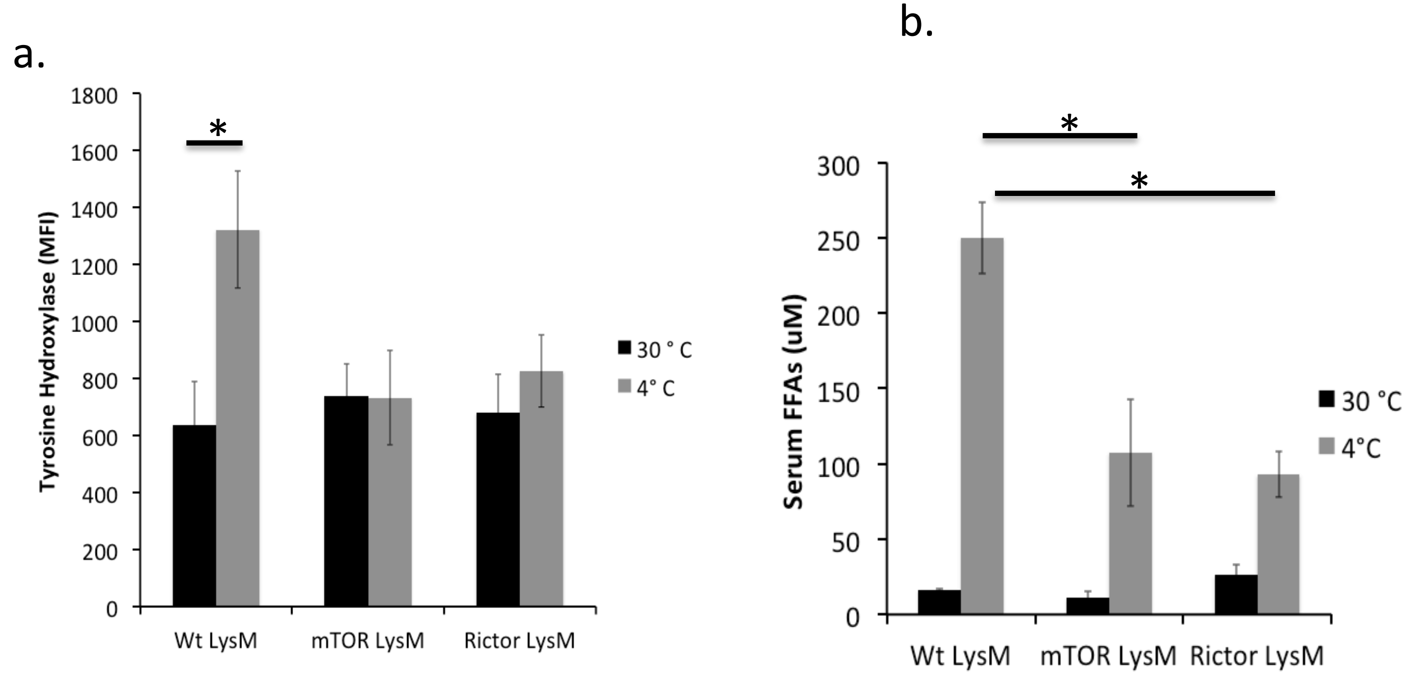

Supplementary Figure 11. mTOR LysM and Rictor LysM mice demonstrate defective lipolysis following cold challenge. a. Flow cytometry of tyrosine hydroxylase expression of white fat macrophages following six-hour cold challenge. **b.** Serum free fatty acid concentration in serum following six-hour cold challenge. Error bars represent standard deviation. Experiments were performed three time with eight mice per group. Statistical significance was determined by either Students t test or 1-way ANOVA followed by Tukeys test. *= statistical significance (p<.05).

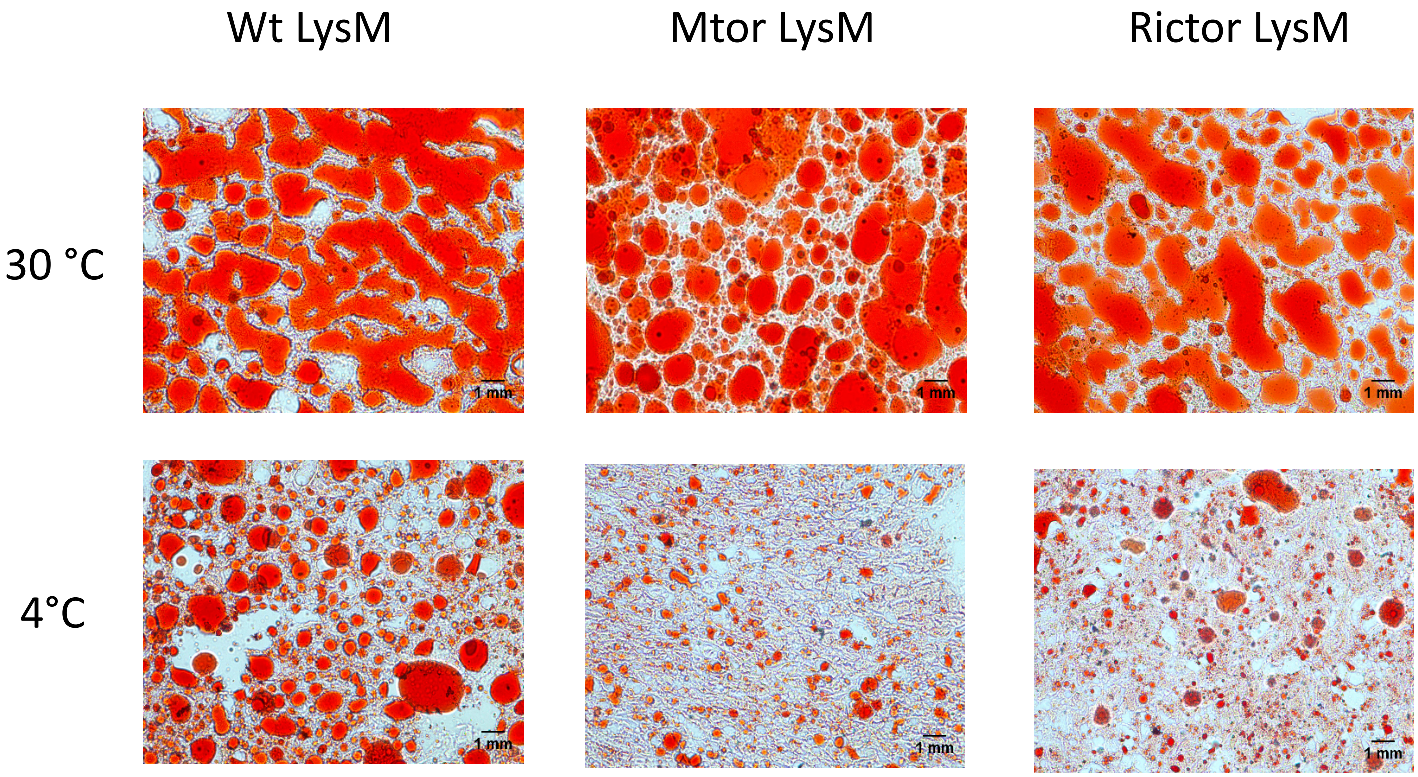

Supplementary Figure 12. Defective thermogenesis by mTOR LysM and Rictor LysM mice is associated with depleted lipids from BAT following cold challenge. Oil red staining of BAT following six-hour cold challenge. Experiments were performed twice with five mice per group.

Antibody	From	Clone	Dilution
CD11b FITC	ebiocience	M1/70	1/500
F4/80 Percp	ebioscience	BM8	1/125
IL4R PE	BD	IL4R-M1	1/500
CD301 Percp	Biolegend	LOM-14	1/125
CD206	Biolegend	Co68C2	1/250
CD11b AF700	BD	M1/70	1/2000
CD11c BV786	BD	HL3	1/200
Siglec F PE-CF594	BD	E50-2440	1/250
CD45 BV510	BD	30-F11	1/1000
Siglec F APC	BD	E50-2440	1/200
CD11c APC	ebioscience	N418	1/500
CD45 APC	ebioscience	30-F11	1/1000
Ly6C APC	BD	AL21	1/500
F4/80 PE	BD	T45-2342	1/500
Ly6G BV421	BD	1A8	1/1000
CD103 APC	BD	M290	1/500
MHC II BV605	BD	M5/114.15.2	1/1000
CD64 PE	BD	X54-5/7.1.1	1/500
CD24 FITC	BD	M1/69	1/500
Tyrosine Hydroxylase	Origene		1/125

Supplementary Figure 13. Table of Antibodies utilized.

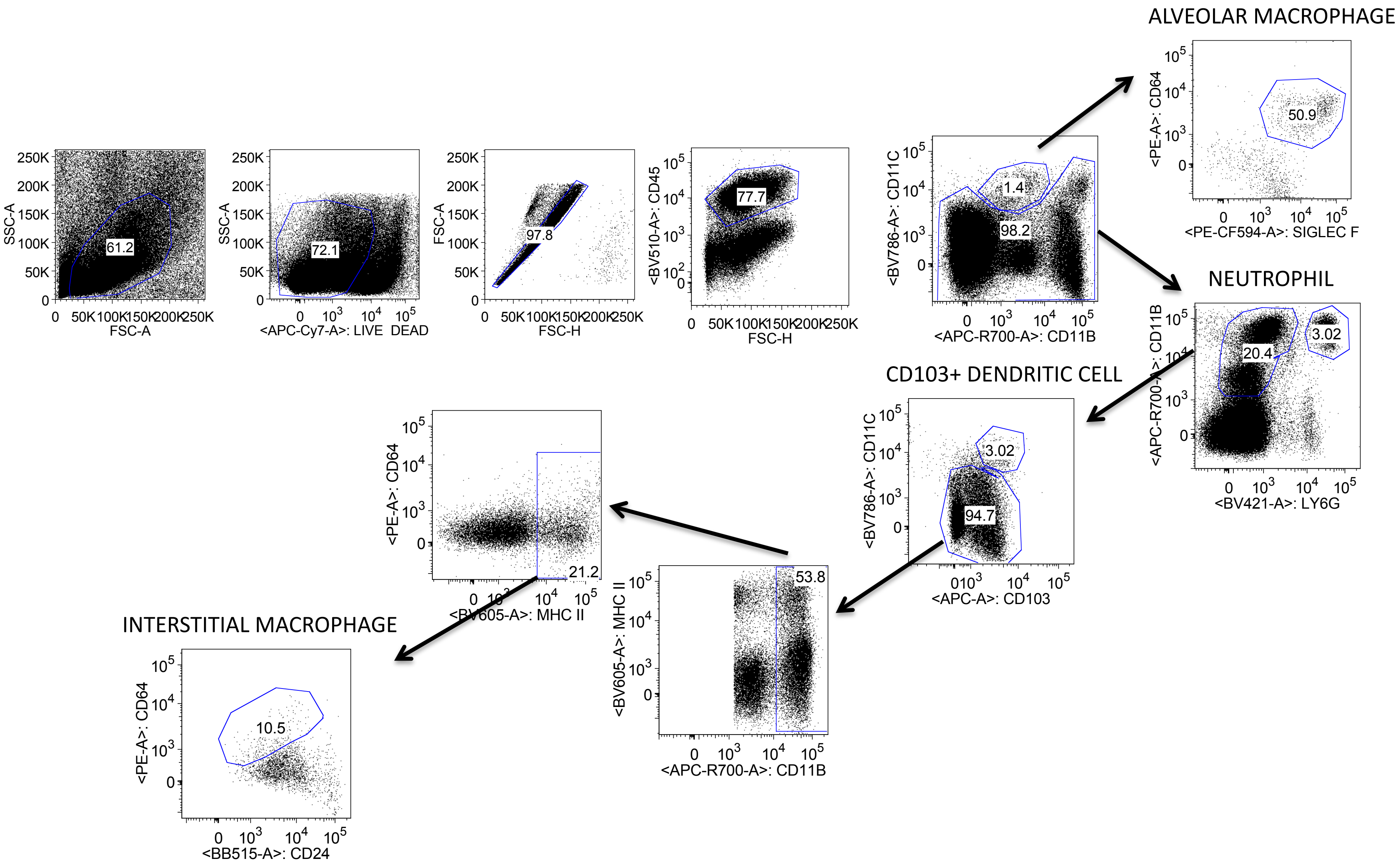

Supplementary Figure 14. Representative FACS gating strategy for lung alveolar macrophages, dendritic cells, neutrophils and interstitial macrophages

Figure 1d.

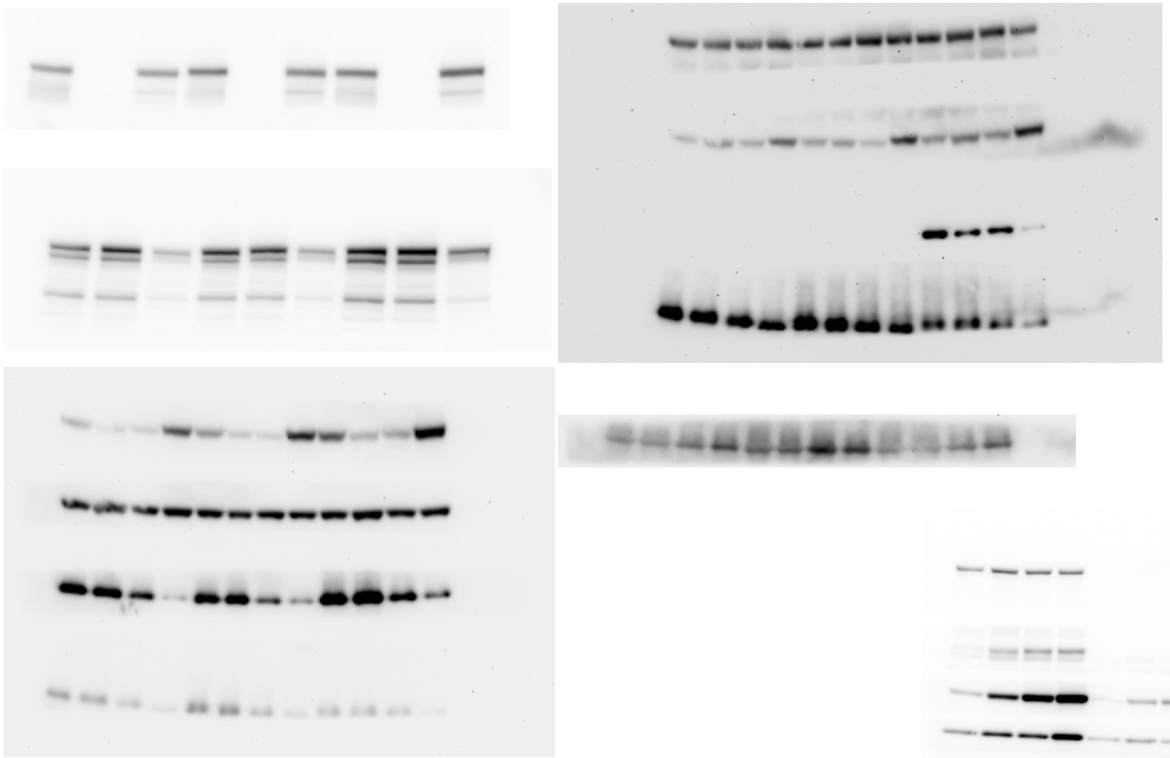

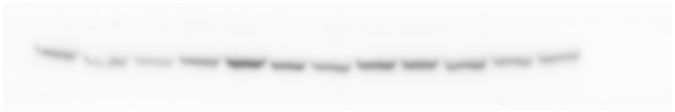

Figure 1e.

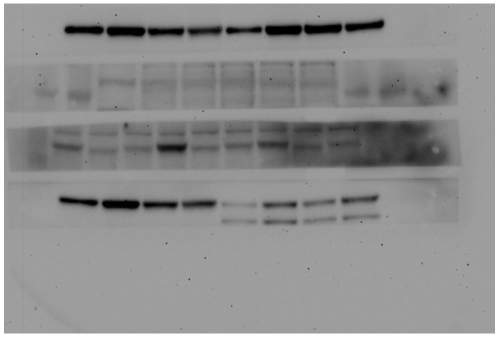

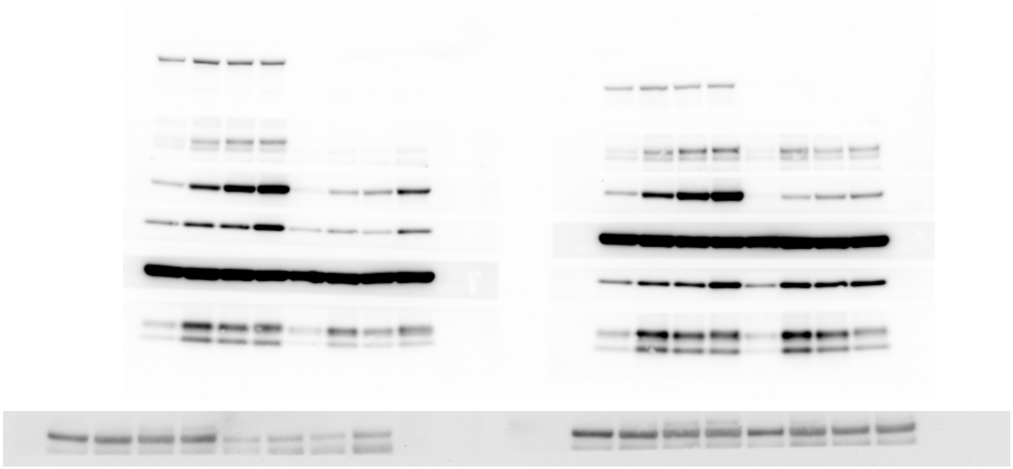

Supplementary Figure 15. Representative western blots for Figure 1.

Figure 3c.

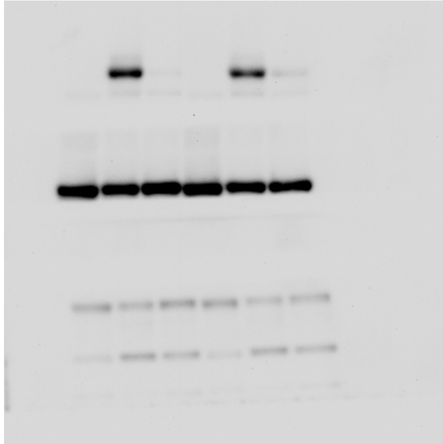

Figure 3e.

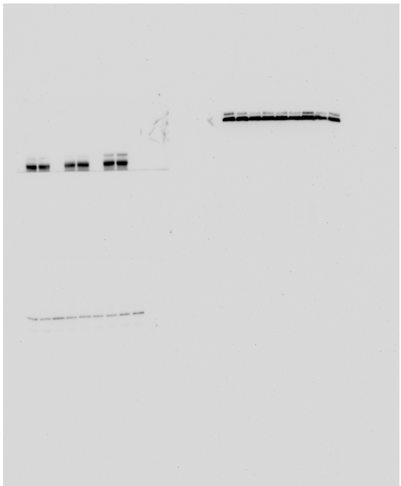

Figure 3f.

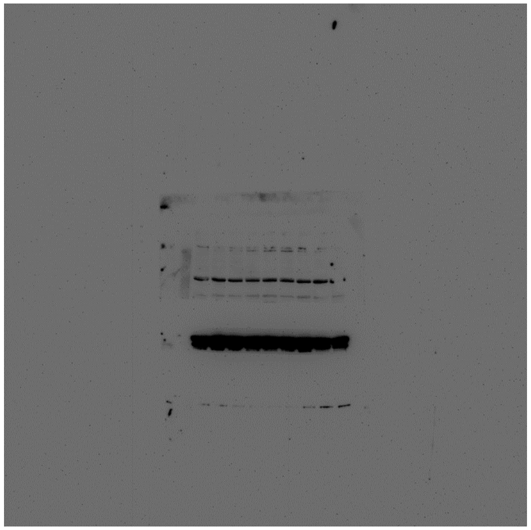

Figure 3g.

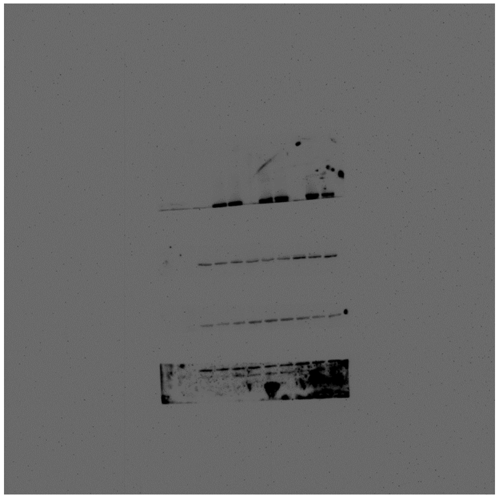

Supplementary Figure 15. Representative western blots for Figure 3.

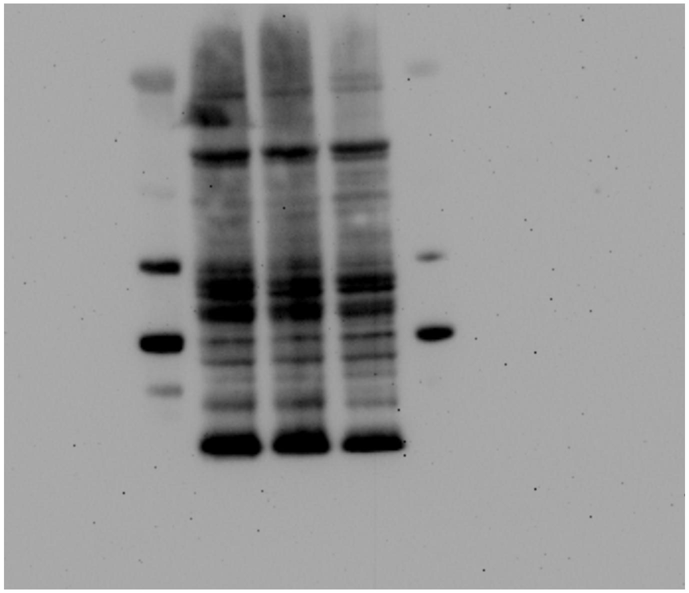

Supplementary Figure 15. Representative western blots for Supplemental figure 10.
